# Cellular Immunity—The Key to Long-Term Protection in Individuals Recovered from SARS-CoV-2 and after Vaccination

**DOI:** 10.3390/vaccines10030442

**Published:** 2022-03-14

**Authors:** Dragan Primorac, Petar Brlek, Vid Matišić, Vilim Molnar, Kristijan Vrdoljak, Renata Zadro, Marijo Parčina

**Affiliations:** 1 St. Catherine Specialty Hospital, 10000 Zagreb, Croatia; petar.brlek@svkatarina.hr (P.B.); vid.matisic@svkatarina.hr (V.M.); vilim.molnar@svkatarina.hr (V.M.); kristijan.vrdoljak3@gmail.com (K.V.); renata.zadro@svkatarina.hr (R.Z.); 2 Medical School, University of Split, 21000 Split, Croatia; 3 Department of Biochemistry & Molecular Biology, The Pennsylvania State University, State College, PA 16802, USA; 4 The Henry C. Lee College of Criminal Justice and Forensic Sciences, University of New Haven, West Haven, CT 06516, USA; 5 Medical School REGIOMED, 96450 Coburg, Germany; 6 School of Medicine, Josip Juraj Strossmayer University of Osijek, 31000 Osijek, Croatia; 7 Medical School, University of Rijeka, 51000 Rijeka, Croatia; 8 Faculty of Dental Medicine and Health, Josip Juraj Strossmayer University of Osijek, 31000 Osijek, Croatia; 9 Medical School, University of Mostar, 88000 Mostar, Bosnia and Herzegovina; 10 National Forensic Sciences University, Gujarat 382007, India; 11 Institute of Medical Microbiology, Immunology and Parasitology (IMMIP), University Hospital Bonn, 53127 Bonn, Germany; parcina@uni-bonn.de

**Keywords:** COVID-19, Omicron variant, Delta variant, breakthrough infection, cellular immunity, infection, vaccination, humoral immunity, SARS-CoV-2

## Abstract

Previous clinical and epidemiological studies have shown that over time antibody titers decrease, and they do not provide long-term mucosa protection against SARS-CoV-2 infection. Additionally, the increase in breakthrough infections that occur more frequently in the vaccinated than in the study participants with previous SARS-CoV-2 infection has recently become a priority public health concern. We measured the amount of interferon-gamma (Quan-T-Cell ELISA) and the level of antibodies (Anti-SARS-CoV-2 QuantiVac ELISA IgG) in the blood of the same patients simultaneously to compare cellular and humoral immunity. A total of 200 study participants (before Omicron variant appearance) were divided into four groups whose levels of cellular and humoral immunity we compared: study participants previously infected with SARS-CoV-2 (group 1); study participants vaccinated with EMA-approved vaccines (group 2); study participants previously infected with SARS-CoV-2, and vaccination history (group 3); and study participants without a history of SARS-CoV-2 infection or vaccination (group 4). Our results showed that study participants who received one of the EMA-approved vaccines and who recovered from COVID-19 (group 3) had significantly higher levels of cellular immunity and antibody titers in comparison with groups 1 and 2. Additionally, we have noticed that the study participants previously infected with SARS-CoV-2 and the study participants vaccinated with EMA-approved vaccines had a long-lasting cellular immunity. Furthermore, antibody levels showed a negative correlation with time since the last contact with a viral antigen, while cellular immunity within 20 months showed as long-term protection. Moreover, out of 200 study participants, only 1 study participant who recovered from COVID-19 (0.5%) was re-infected, while a total of 6 study participants (3%) were infected with SARS-CoV-2 after receiving the vaccine. This study suggests that cellular immunity—unlike humoral immunity, thanks to memory T cells—represents long-term protection in individuals recovered from SARS-CoV-2 and after vaccination.

## 1. Introduction

Multiple epidemiological and clinical studies, including studies during the recent period of variants of concern (VOC) transmission, showed that previous SARS-CoV-2 infection induced a long-lived humoral immune memory in patients who became equally protected as those vaccinated [1]. The emergence of breakthrough infections with SARS-CoV-2 has raised concerns about the durability of vaccine effectiveness, especially against the Delta and the Omicron variants [2,3,4]. Since most vaccines are based solely on the viral S antigen, it is worrisome that new VOC, such as the Omicron variant with 33 uniquemutations in the spike protein, could potentially bypass the humoral immune response [5,6,7,8]. Moreover, although plasma-neutralizing antibody titers may predict some level of protection against symptomatic infection, the duration of that protection remains unclear [3].

Recent work suggests that cellular immunity plays an important role in addition to humoral immunity and, with the help of memory cells, represents long-term immune protection against severe disease [3,9]. Previous findings suggest that both CD4+ and CD8+ T cells coordinate SARS-CoV-2-specific adaptive immune responses in COVID-19, which is associated with milder disease [10]. Furthermore, recent papers showed that an imbalance in Th1- and Th17-related cytokines (IFN-γ and IL-17) was associated with significantly increased mortality from COVID-19, suggesting the important role of cellular immunity in patient outcomes during SARS-CoV2 infection [11]. The presence of cellular immunity also stands out in a group of patients lacking humoral immunity (immunosuppressed patients, patients without detectable SARS-CoV-2 IgG to S1 protein after vaccination, patients with agammaglobulinemia) [12,13,14,15].

Our study aimed to compare immune responses to the viral S antigen in a cohort vaccinated against and/or recovered from the SARS-CoV-2 infection at different time points in order to determine the longevity of the cellular immune response and to compare it to that of humoral immunity.

## 2. Materials and Methods

The study included 200 participants tested at St. Catherine Specialty Hospital. All study participants filled out a detailed questionnaire on previous SARS-CoV-2 infection and/or vaccination as well as re-infection and they provided a venous blood sample for the detection of a cellular and a humoral immune response. Through the questionnaire, we also collected data on the number of symptoms study participants experienced during COVID-19 infection and/or vaccination, and the time since their last contact with the viral S antigen to correlate with cellular and humoral immune levels.

The Ethics committee of St. Catherine Specialty Hospital approved the study. All participants provided written informed consent.

### 2.1. Cohorts

Study participants were divided into four groups: study participants with a previous SARS-CoV-2 infection (group 1); study participants vaccinated with an EMA-approved vaccine (Pfizer/BioNTech, Moderna, AstraZeneca, or Johnson & Johnson) (group 2); study participants with a previous SARS-CoV-2 infection and a vaccination history (group 3); and study participants without a history of SARS-CoV-2 infection or a vaccination (group 4). Cohorts were defined according to the results of a PCR, rapid antigen test, and they were further verified by the physician who took the study participant’s history. In the group of vaccinated study participants, a total of 90.9% of participants were fully vaccinated with two doses, or one dose in the case of the Johnson & Johnson vaccine. Study participants from group 4 were not tested with a PCR test due to their medical history according to which they had never had a SARS-CoV-2 infection. Additionally, they were tested by the Anti-SARS-CoV-2 QuantiVac ELISA IgG test to exclude previous infections.

### 2.2. Analysis of Cellular and Humoral Immunity

For the analysis of cellular immunity, the Quan-T-Cell SARS-CoV-2 in combination with the Quan-T-Cell ELISA (Euroimmun Medizinische Labordiagnostika, Luebeck, Germany) was used. The principle of the test is a measurement of interferon-gamma released by activated immune cells. Fresh whole blood samples were collected in heparinized tubes and pipetted into the three stimulation tubes (Quan-T-Cell SARS-CoV-2): (1) COV-2 IGRA (interferon-gamma release assay) Blank was used for measuring individual interferon-gamma concentrations as it contained no activating components; (2) CoV-2 IGRA Tube was coated with peptide components of the S1 domain of the SARS-CoV-2 spike protein; and (3) CoV-2 IGRA Stim was coated with mitogen to verify if the sample contained a sufficient number of viable and functional T cells. After incubation of the individual whole blood in the stimulation tubes for 20–24 h at 37 °C, the separated plasma was used to determine interferon-gamma concentration by Quan-T-Cell ELISA. Anti-SARS-CoV-2 QuantiVac ELISA IgG (Euroimmun Medizinische Labordiagnostika, Luebeck, Germany) was used to quantitatively determine human antibodies of the immunoglobulin class IgG against the S1 domain of the SARS-CoV-2 spike protein in the sera of investigated individuals. Antibody titer was measured from the same blood sample to compare humoral and cellular immunity.

The cut-off value was 200 mIU/mL for cellular immunity (interferon-gamma level) and 35.2 IU/mL for antibody level, and all values below were reported as negative results [16].

### 2.3. Statistical Analysis

We performed statistical analysis in the software package IBM SPSS Statistics 23.0 (SPSS, Chicago, IL, USA), with a significance level of *p* < 0.05. The normality of the distribution of individual parameters within the groups was tested using the Kolmogorov–Smirnov and the Shapiro–Wilk tests of normality. Since the analysis showed a non-normal distribution of data, non-parametric statistical tests (Kruskal–Wallis, Mann–Whitney, and Spearman correlation test) were used. The Kruskal–Wallis test showed whether there were differences in the measured values of cellular and humoral immunity between three or more groups. The Mann–Whitney test was used to compare individual groups. Cellular and humoral immunity, as well as study participants’ ages, number of symptoms, and the time since the last contact with the SARS-CoV-2 antigen were correlated by performing the Spearman correlation test.

## 3. Results

### 3.1. Population Characteristics

Analyzed data obtained from 200 study participants are shown in Table 1. Out of 200 study participants, only 1 study participant (0.5%) was symptomatically re-infected with SARS-CoV-2 (confirmed by PCR test), while 6 study participants (3%) became symptomatically infected after vaccination, which was also confirmed with PCR testing. PCR testing was done before or after inclusion in our study. However, the re-infected patient and the patients infected after vaccination had mild clinical symptoms of COVID-19, and they were not hospitalized.

Of the total number of study participants, 55 were diagnosed with SARS-CoV-2 infection (group 1). Furthermore, 55 study participants were vaccinated (group 2), while 45 were not vaccinated and had not recovered from SARS-CoV-2 infection (group 4). The remaining study participants (45) had previously recovered from infection and were vaccinated against SARS-CoV-2 (group 3).

### 3.2. Levels of Cellular and Humoral Immunity

**Figure 1:**
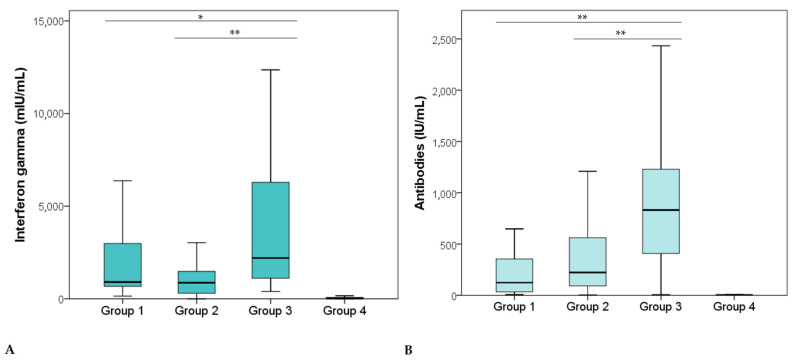
Level of cellular (**A**) and humoral (**B**) immunity in study participants with a previous SARS-CoV-2 infection (group 1), study participants vaccinated with one of the SARS-CoV-2 vaccines (group 2), study participants who had a past SARS-CoV-2 infection and a vaccination history (group 3), and study participants without a history of SARS-CoV-2 infection or a vaccination (group 4). *—*p* < 0.05 (Mann–Whitney); **—*p* < 0.001 (Mann–Whitney).

We analyzed data obtained from 102 (51.0%) male study participants and 98 (49.0%) female study participants. There was neither a significant difference in the level of cellular immunity between males and females (*p* = 0.277) nor any difference in antibody levels between males and females (*p* = 0.281). The analysis showed no significant difference in the level of cellular immunity between groups 1 and 2 (*p* = 0.050). The Mann–Whitney test showed significantly higher cellular immunity in group 3 compared to group 1 (*p* = 0.005) and group 2 (*p* < 0.001) (Figure 1A). All study participants in group 4 had a negative result for the cellular immune response (<200 mIU/mL). Furthermore, we observed significantly higher antibody levels in group 3 compared to group 1 (*p* < 0.001) and group 2 (*p* < 0.001) (Figure 1B). These results suggest that individuals who have a combination of infection and vaccination have significantly higher levels of both humoral and cellular immunity to SARS-CoV-2 antigen S compared to study participants with a previous SARS-CoV-2 infection or study participants only vaccinated against SARS-CoV-2.

The Spearman test showed a significant positive correlation (r = 0.801) between cellular immunity and antibody levels (*p* < 0.001), as well as a positive correlation between cellular (r = 0.400, *p* < 0.001) and humoral immunity (r = 0.314, *p* < 0.001) in comparison with the number of symptoms (Table 2). Additionally, the Spearman test showed a significant negative correlation (r = −0.426) between antibody levels and the time since the last contact with the viral antigen S (*p* < 0.001), while no significant correlation existed for cellular immunity (*p* = 0.240). Moreover, our results show a statistically significant positive correlation between the immune response (cellular and humoral) with the age of study participants. Table 2 shows the correlation tests with the corresponding coefficients and *p* values.

**Figure 2:**
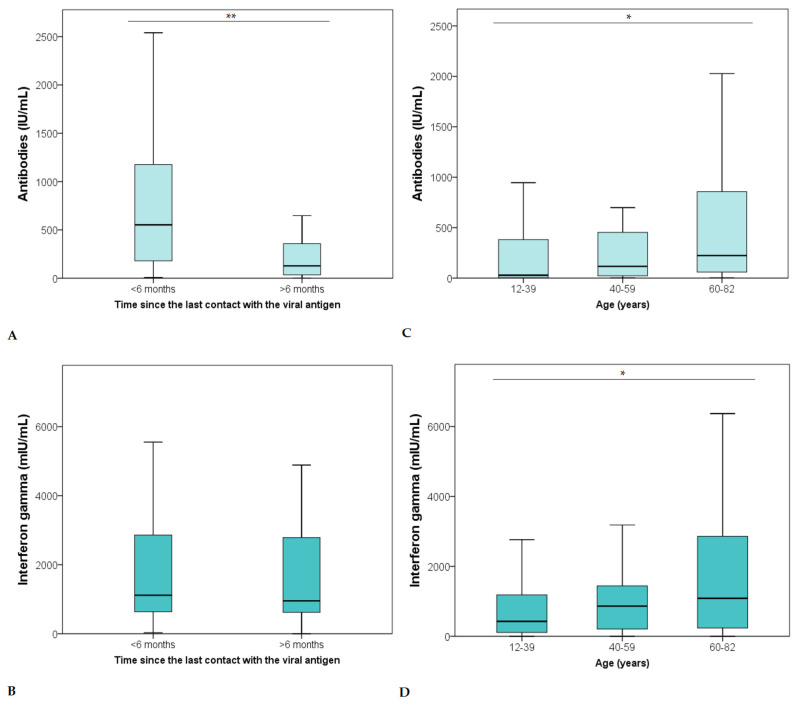
Level of humoral (**A**) and cellular (**B**) immunity in study participants exposed to the viral antigen less than six months prior than those exposed more than six months prior. Levels of humoral (**C**) and cellular (**D**) immunity in different age groups show age-dependent differences in distribution. In Figure 2A,B, study participants were divided into two groups, with an arbitrary limit of 6 months. *—*p* < 0.05 (Mann–Whitney); **—*p* < 0.001 (Mann–Whitney).

The Mann–Whitney test showed significantly higher antibody levels in study participants exposed to the viral antigen less than six months prior than those exposed more than six months prior (*p* < 0.001) (Figure 2A). In contrast, levels of cellular immunity were not significantly different when comparing time elapsed from exposure to the SARS-CoV-2 antigen S (*p* = 0.483) (Figure 2B).

Furthermore, we observed significantly higher antibody levels in study participants between 60 to 82 years old in comparison with those between 12 to 39 years old (*p* = 0.002) (Figure 2C). The same significant difference was observed for cellular immunity (interferon-gamma level) (Mann–Whitney test, *p* = 0.014) (Figure 2D).

## 4. Discussion

Our results, determined by measuring interferon-gamma levels, showed that cellular immunity provided long-term protection, while at the same time measurements of humoral immunity (antibody levels) showed a decrease over time. Moreover, four study groups showed distinct differences in the level of cellular and humoral immunity. However, the level of cellular immunity in the vaccinated group was equal to that of study participants previously infected with SARS-CoV-2 (group 1). A recent study showed that the third dose of the vaccine in adults aged 60 years and older was associated with significantly elevated IgG titers, noting that the IgG response correlates with disease protection [17]. However, Kojima and Klausner stated that antibodies are incomplete predictors of protection against SARS-CoV-2 [1]. Our results showed a significant decrease in antibodies six months after the last contact with the SARS-CoV-2 antigen S. It is important to note that vector- or mRNA-based vaccination also stimulates the cellular immune response. Our results showed no significant difference in the interferon-gamma levels between the vaccinated and the COVID-19 recovered study participants, while simultaneously showing a statistically significant decrease in antibody titers six months after contact with the viral antigen S. These findings suggest that cellular response measurement provides data that represents a more uniform method for assessing immunity levels in the general population.

Le Bert et al. showed that patients who recovered from severe acute respiratory syndrome (SARS) possess long-lasting memory T cells that are reactive to the N protein of SARS-CoV 17 years after the outbreak of SARS in 2003 and that these T cells displayed cross-reactivity to the N protein of SARS-CoV-2 [18]. Moreover, SARS-CoV infection also caused the formation of memory B cells that lasted six years after the infection and showed a shorter survival period compared with the CD8+ T cells [18,19,20]. In cases of mutations occurring in VOC, recent data has shown that memory mediated by CD4+ T cells in recovered and vaccinated SARS-CoV-2 patients has a better-defending capability in comparison with the neutralizing antibody function and that cross-reactive SARS-CoV-2-specific T cell immunity presumably plays a role in protecting against these VOC [21,22,23,24,25].

Such findings indicate a potentially “hidden” role for cellular immunity during the current pandemic about which we do not know enough. The results of this study demonstrate a sustained cellular immune response that does not decrease in the 20 month-period after the last contact with the SARS-CoV-2 S antigen, which indicates that, for now, cellular immunity represents a long-lasting immune response against SARS-CoV-2 infection compared to the waning antibody titers for patients who have recovered from SARS-CoV-2 and those who were vaccinated. Moreover, our results show significantly higher levels of both cellular and humoral immunity in patients that were both vaccinated and recovered from SARS-CoV-2 compared to those who were only vaccinated or only recovered from SARS-CoV-2.

Nevertheless, in our study, we noticed that out of 200 study participants, only 1 study participant (0.5%) was re-infected, which coincides with the results of Vitale et al., who showed 5 re-infections in the cohort of 1579 patients (0.31%) [26]. Patients who have recovered from SARS-CoV-2 presumably acquire cellular immunity to various viral antigens, while the vaccine creates immunity only against the spike protein, which may be the reason for the significantly reduced reinfection rate in patients who have recovered from SARS-CoV-2. However, we could not confirm or dismiss this presumption, as we did not test the immune response to other viral antigens [27,28,29,30]. Due to the importance of T-cell immunity, the development of a new vaccine that will primarily stimulate cellular immunity has begun. CoVac-1, a COVID-19 vaccine candidate designed to induce cellular immunity activated a T-cell response that exceeded those induced by SARS-CoV-2 infection and approved vaccines [31]. These new findings on the durability of the cellular immune response to COVID-19 as well as new treatment strategies, including therapy with mesenchymal stem cells, will shed light on novel therapeutic possibilities in the COVID-19 pandemic [32].

Study limitations include using the S antigen-only cytokine release assay which may not detect markers of cellular immunity from other antigens that could differ in intensity or duration. This study was conducted before the appearance of the Omicron variant in our population. Moreover, in the vaccinated cohort, we detected only re-infected patients who were symptomatic and therefore confirmed by PCR testing. The actual number of contacts with the virus in the population could be proven by analyzing the immune response to SARS-CoV-2 antigen N. However, due to the unavailability and the insufficient validation of such tests, we decided not to measure cellular immune reactions with N-peptide IGRA-assays.

## 5. Conclusions

This study demonstrates the importance of the cellular immune response measured by interferon-gamma and its potential broad clinical application. Our results show that the measurement of interferon-gamma is a clear and a long-term indicator of the state of the cellular immune response not only in the vaccinated but also in patients recovering from SARS-CoV-2 infection. In addition, we have noticed that the study participants previously infected with SARS-CoV-2 and the study participants vaccinated with EMA-approved vaccines had a long-lasting cellular immunity. Forthcoming studies based on measuring cellular immunity will impact the understanding of not only the COVID-19 pandemic but also other potential pandemics.

## Figures and Tables

**Table 1:** The data show the levels of cellular immunity, humoral immunity (antibodies), and the distribution of the age and the sex of study participants within the study groups.

		Group 1 (N = 55)	Group 2 (N = 55)	Group 3 (N = 45)	Group 4 (N = 45)
Cellular immunity (mIU/mL)	MD	932.0	866.0	2203.0	22.0
	IQR	2514.0	1242.0	5556.0	78.5
Antibodies (IU/mL)	MD	128.0	222.9	831.7	3.3
	IQR	320.4	470.9	906.6	2.8
Age (years)	MD	46.0	52.0	49.0	43.0
	IQR	15.0	13.0	20.0	14.0
Sex, No. (%)	M	58.2	52.7	48.9	42.2
	F	41.8	47.3	51.1	57.8

MD—median; IQR—interquartile range; M—male; F—female.

**Table 2:** Correlation table between cellular immunity, antibodies titer, study participant’s age, the time since the last contact with the viral antigen, and the number of symptoms study participants experienced.

		Cellular Immunity (mIU/mL)	Antibodies (IU/mL)	Age (Years)	Time (Months)	Number of Symptoms
Cellular immunity (mIU/mL)	r	1.000	0.801 **	0.189 **	−0.095	0.400 **
	*p*	.	<0.001	0.007	0.240	<0.001
	N	200	200	200	155	200
Antibodies (IU/mL)	r	0.801 **	1.000	0.225 **	−0.426 **	0.314 **
	*p*	<0.001	.	0.001	<0.001	<0.001
	N	200	200	200	155	200
Age (years)	r	0.189 **	0.225 **	1.000	0.073	−0.024
	*p*	0.007	0.001	.	0.365	0.731
	N	200	200	200	155	200
TIME (months)	r	−0.095	–0.426 **	0.073	1.000	0.182 *
	*p*	0.240	<0.001	0.365	.	0.023
	N	155	155	155	155	155
Number of symptoms	r	0.400 **	0.314 **	−0.024	0.182 *	1.000
	*p*	<0.001	<0.001	0.731	0.023	.
	N	200	200	200	155	200

r—Spearman’s correlation coefficient; N—sample size; TIME- the time since the last contact with the viral antigen; *—correlation is significant at the 0.05 level (2-tailed); **—correlation is significant at the 0.01 level (2-tailed).

## Data Availability

The data sets generated during this study are available from the corresponding author on request.
